# Association Between Computed-Tomography-Derived Erector Spinae Muscle Characteristics and Swallowing Outcomes in Older Patients with Pneumonia

**DOI:** 10.3390/healthcare14162461

**Published:** 2026-08-09

**Authors:** Yuichi Murakawa, Akira Tamaki, Ryota Matsuzawa, Shinjiro Miyazaki, Koji Fujishima, Tatsuma Hori, Miki Naide, Kenichiro Sakai, Tomoya Ishii

**Affiliations:** 1 Graduate School of Rehabilitation Science, Hyogo Medical University, 1-3-6 Minatojima, Kobe 650-8530, Hyogo, Japan; dhe2505@hyo-med.ac.jp (Y.M.);; 2 Department of Rehabilitation Technology, Sanuki Municipal Hospital, 387-1 Ishida Higashi Kou, Sanuki City 769-2393, Kagawa, Japan; 3 Rehabilitation Center, KKR Takamatsu Hospital, 4-18 Tenjinmae, Takamatsu City 760-0018, Kagawa, Japan; 4 Department of Respiratory Medicine, Sanuki Municipal Hospital, 387-1 Ishida Higashi Kou, Sanuki City 769-2393, Kagawa, Japan

**Keywords:** pneumonia, erector spinae muscle, muscle mass, muscle quality, swallowing outcome

## Abstract

**Highlights:**

**What are the main findings?**

- In older patients with pneumonia, the muscle mass of the erector spinae muscles (ESM), as measured using computed tomography (CT) scans, is associated with swallowing function at discharge.
- In older patients with pneumonia, the muscle quality of the ESM, as measured using computed tomography (CT) imaging, is not associated with swallowing function at discharge.

**What are the implications of the main findings?**

- Measuring the muscle mass of the ESM may be useful for assessing swallowing function in older patients with pneumonia at the time of discharge.
- ESM should be considered not as an alternative to direct swallowing assessment, but as a potential complementary marker when CT images are already available.

**Abstract:**

Background/Objectives: Trunk muscle mass and quality may be related to swallowing function; however, it is unclear whether the same trend is observed in older patients with pneumonia. We aimed to determine the impact of the mass and quality of the erector spinae muscles (ESM), measured using chest computed tomography (CT), on swallowing function decline during hospitalization. Methods: The participants were patients hospitalized at Sanuki Municipal Hospital with a diagnosis of pneumonia who underwent pulmonary rehabilitation (PR). A total of 450 patients met the eligibility criteria and were included in the analysis. The cross-sectional area of the ESM normalized by body surface area (ESM_CSA_/BSA) was measured from chest CT images as an indicator of muscle mass, whereas the CT attenuation value of the ESM (ESM_CT_) was used as an indicator of muscle quality. The primary outcome was the Functional Oral Intake Scale (FOIS) score at discharge. Results: Patients with a FOIS score of 6–7 at discharge exhibited significantly higher ESM_CSA_/BSA values than those with lower FOIS scores. In ordinal logistic regression analysis, ESM_CSA_/BSA was independently associated with the FOIS score at discharge (odds ratio, 1.09; 95% confidence interval, 1.03–1.15; *p* = 0.004). However, this association was no longer significant after adjustment for the Functional Independence Measure score at the start of PR. In contrast, ESM_CT_, an indicator of muscle quality, was not associated with FOIS score at discharge. Conclusions: In older patients with pneumonia, ESM may reflect overall physical function, which should be considered when interpreting its association with swallowing outcomes.

## 1. Introduction

Recently, Japan’s population has been aging more rapidly than that of other countries. There is a close relationship between the aging of the population and the incidence of pneumonia [1]. Many older patients with pneumonia reportedly suffer from aspiration pneumonia [2], making it important to understand their swallowing function. The rate of regaining oral intake at discharge among patients with aspiration pneumonia is 75.9% [3] or 65.0% [4], suggesting that many patients still exhibit swallowing dysfunction at the time of discharge. Insufficient recovery of swallowing function at discharge makes it difficult for older patients with pneumonia to return to their previous residence, increases their dependence on medical care, and places a greater burden on caregivers. Furthermore, the nutritional pathway at hospital discharge is associated with life expectancy in older patients with aspiration pneumonia [4]; hence, it is essential to understand factors related to swallowing function during hospitalization in order to implement therapeutic interventions and prevent its decline.

Many factors associated with swallowing function have been reported, including body mass index (BMI) [5], low body weight [6], sex [6], severity of illness [6], comorbidities [6], duration of fasting [7], activities of daily living (ADL) performance [5,6,8], performance status [9], mobility assistance [9], malnutrition [9], decreased nutritional intake [9], and length of hospital stay [9].

Among these factors, the relationship between nutritional status and swallowing function has been the focus of attention. A study investigating the relationship between muscle mass and swallowing function found that a decrease in skeletal muscle index [5,8] and the presence of sarcopenia [8] were associated with swallowing function. Furthermore, a study investigating the relationship between muscle quality and swallowing function found that quadriceps muscle brightness was associated with swallowing function [10]. For these reasons, it is essential to evaluate skeletal muscles, which are considered important in nutritional status, to understand swallowing function. Recently, methods have been developed to measure the quantity and quality of skeletal muscle using computed tomography (CT) images. In respiratory diseases, associations between chest CT-derived parameters and prognosis have been reported in conditions such as chronic obstructive pulmonary disease [11] and idiopathic pulmonary fibrosis [12]. As such, it has been shown that a decrease in skeletal muscle mass and sarcopenia are associated with dysphagia in older adults. However, it remains unclear whether skeletal muscle characteristics derived from CT images are associated with outcomes in older patients with pneumonia.

Recently, we reported that muscle mass rather than muscle quality of the erector spinae muscles (ESM) measured from chest CT images may be associated with short-term life expectancy in older patients with aspiration pneumonia [13]. In respiratory diseases, chest CT images are often obtained in daily clinical practice to assess disease pathophysiology and can be used to measure skeletal muscle mass and muscle quality.

A study investigating the impact of muscle mass and quality, measured using chest CT imaging, on swallowing function in older hospitalized patients found that skeletal muscle index and intramuscular adipose tissue of the trunk were associated with swallowing function [14]. These findings suggest that trunk muscle mass and muscle quality may be related to swallowing function. In particular, the ESM is involved in trunk stability, respiratory mechanics, and maintenance of posture, all of which are considered to be related to swallowing function. However, it remains unclear whether this association is also observed in older patients with pneumonia.

Our group previously reported that computed-tomography-derived erector spinae muscle characteristics were associated with functional decline in older patients with pneumonia [15]. However, whether these muscle characteristics are associated with swallowing outcomes remains unclear. Given the high prevalence of dysphagia among older patients with pneumonia and its substantial impact on nutritional status, aspiration risk, and clinical recovery, further investigation is warranted.

Therefore, we hypothesized that lower ESM_CSA_/BSA and ESM_CT_ would be associated with poorer swallowing outcomes in older patients with pneumonia. The purpose of this study was to clarify the impact of ESM muscle mass and quality, as measured using chest CT imaging, on the decline in swallowing function during hospitalization in older patients with pneumonia. If validated, this study will aid pneumonia treatment by enabling the prediction of swallowing function decline—an essential parameter in older patients with pneumonia—based on parameters measured using chest CT during hospitalization.

## 2. Materials and Methods

### 2.1. Study Design and Participants

This single-center, retrospective, observational study included patients admitted to Sanuki Municipal Hospital with a diagnosis of pneumonia who underwent pulmonary rehabilitation (PR) from April 2018 to March 2024. Exclusion criteria were as follows: (1) age younger than 65 years; (2) in-hospital death; (3) transfer from another hospital or transfer during hospitalization; (4) bedridden status before hospitalization; and (5) missing or unmeasured chest CT data at admission. The diagnosis of pneumonia at Sanuki Municipal Hospital was based on clinical symptoms (fever, sputum, and cough), coarse crackles on auscultation, elevated white blood cell (WBC) and C-reactive protein (CRP) levels, findings on chest radiographs, and chest CT images. Patients received PR according to routine clinical practice at our institution. PR was initiated after referral by the attending physician and focused on early mobilization, physical conditioning, and improvement of ADL. Resistance training was introduced when clinically appropriate, considering the patient’s general condition and recovery from pneumonia. PR was individualized based on each patient’s physical status and tolerance. One session typically lasts between 20 and 40 min. A portion of the study cohort overlapped with that of our previous publication investigating functional decline; however, the present study addressed a different research question focusing on swallowing outcomes and utilized different outcome measures and analytical approaches. 

Although participants in this study did not provide written or verbal informed consent, they were allowed to refuse or withdraw from participation at any time. The opt-out procedure conforms to the Japanese Ethical Guidelines for Epidemiological Research for observational studies that use existing data. The study protocol, including the consent procedure (informed consent waiver), was approved by the Ethics Review Board of Hyogo Medical University (approval number: 5058) and conducted in accordance with the principles of the Declaration of Helsinki.

### 2.2. Measurements

Characteristics of each participant, including age, sex, BMI, Charlson Comorbidity Index (CCI), residence location before admission, A-DROP score, mechanical ventilation, intravenous antibiotic administration, WBC, CRP, albumin (Alb), hemoglobin (Hb), Functional Independence Measure (FIM) at the start of PR and at discharge, days to start PR, days to start oral or enteral nutrition, length of hospital stay, and residence location after discharge were collected retrospectively from the medical records. Based on previous studies [16], the CCI includes 19 weighted conditions: myocardial infarction, congestive heart failure, peripheral vascular disease, cerebrovascular disease, dementia, chronic pulmonary disease, connective tissue disease, peptic ulcer disease, mild liver disease, diabetes, hemiplegia, moderate or severe renal disease, diabetes with end organ damage, any tumor, leukemia, lymphoma, moderate or severe liver disease, metastatic solid tumor, and AIDS. The A-DROP score is based on age (male ≥ 70 years, female ≥ 75 years), dehydration (blood urea nitrogen ≥ 21 mg/dL), respiratory status (peripheral oxygen saturation ≤ 90% or partial pressure of arterial oxygen ≤ 60 mmHg), orientation disturbance, and blood pressure (systolic blood pressure ≤ 90 mmHg) [17]. The day of initiation of PR was defined as the day when a physical or occupational therapist first initiated PR after admission. The day of initiation of oral intake or enteral nutrition was defined as the day on which nutritional management via oral intake or enteral nutrition was started. The FIM was calculated based on 13 motor items and 5 cognitive items, each scored from 1 to 7 according to the level of assistance required, yielding a total score ranging from 18 to 126. 

Chest CT images taken at admission were used to measure muscle mass and muscle quality. SYNAPSE 5 (Version 5.5, FUJIFILM Medical Co., Ltd., Tokyo, Japan) was used for analysis. The left and right ESMs at the level of the lower 12th thoracic vertebra were measured using the manual tracing method, following previous studies [13,15,18,19]. The cross-sectional area (CSA) of the left and right ESMs was defined as muscle mass (ESM_CSA_), and the CT values of the left and right ESMs as muscle quality (ESM_CT_). ESM_CSA_ was adjusted for body surface area (ESM_CSA_/BSA). ESM_CT_ was calculated by averaging the CT values of the left and right ESMs. Studies have verified the validity of the ESM using CT, with intraclass correlation coefficients (ICC) for intra-rater and inter-rater reliability of 0.972 and 0.970, respectively [20].

### 2.3. Clinical Outcomes

The Functional Oral Intake Scale (FOIS) was used to determine the clinical outcome of swallowing function [21]. The FOIS is a standardized seven-level assessment of tube feeding to oral intake developed by Crary et al. The higher the level, the better the swallowing function. FOIS before admission and at discharge was determined by the type of meals the patients consumed at those times. 

### 2.4. Statistical Analysis

First, the study participants were classified into three groups based on their FOIS scores at discharge: the Poor FOIS group (FOIS: 1–3), the Moderate FOIS group (FOIS: 4–5), and the Good FOIS group (FOIS: 6–7) [22]. Subsequently, the characteristics of the participants were compared using the Kruskal–Wallis test and the chi-square test. Next, we used Spearman’s rank correlation coefficient to examine the relationship between ESM_CSA_/BSA, ESM_CT_, and the FOIS score at discharge. Next, we performed an ordinal logistic regression analysis with FOIS score at discharge as the dependent variable. ESM_CSA_/BSA and ESM_CT_ were included as explanatory variables, and age, sex, BMI, CCI, A-DROP score, CRP, Alb, intravenous antibiotic administration, FOIS score before hospitalization, and FIM score at the start of PR were included as covariates. Covariates were selected based on the previous literature and clinical relevance to swallowing outcomes in older patients with pneumonia [5,6,7,8,9]. In addition, we conducted a stratified analysis to investigate the impact of differences in patient characteristics on the association between ESMCSA/BSA and FOIS at discharge ≥6. The stratified analysis (divided into two groups based on the median) classified patients by age (85 years or older, under 85 years), sex (male, female), A-DROP score (2 or higher, less than 2), and Alb (3.32 g/dL or higher, less than 3.32 g/dL). Finally, we conducted a sensitivity analysis limited to patients with a pre-hospitalization FOIS score ≥ 6. Patients were classified into two groups based on FOIS score ≥ 6 at discharge, and between-group comparisons were performed using the Mann–Whitney U test for continuous variables and the chi-square test for categorical variables. Subsequently, we performed a multiple logistic regression analysis with FOIS at discharge as the dependent variable, and ESM_CSA_/BSA and ESM_CT_ as predictors, adjusting for the same covariates listed above. Multicollinearity was assessed using the variance inflation factor (VIF), with values < 5 considered acceptable. Odds ratios (ORs) and 95% confidence intervals (CIs) were calculated. Statistical analyses were performed using EZR version 1.68 (Saitama Medical Center, Jichi Medical University, Saitama, Japan) [23], and a two-sided significance level of 5% was applied to all tests.

## 3. Results

**Figure 1:**
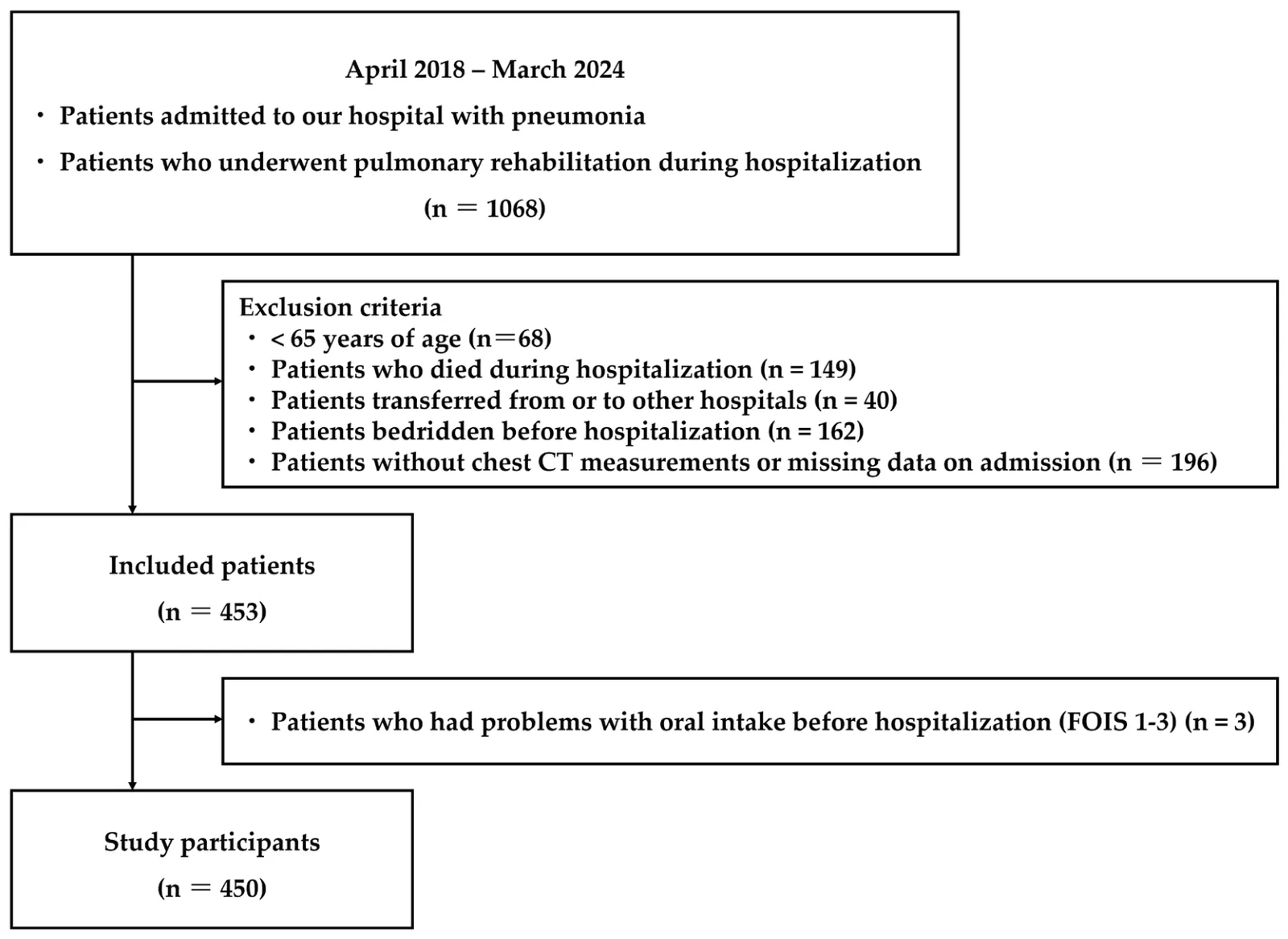
Flow chart of the inclusion of study participants. CT, computed tomography; FOIS, functional oral intake scale.

This study included 453 participants out of 1068 who met the inclusion criteria. Furthermore, 450 participants were analyzed after excluding one individual who had difficulty with oral intake before hospitalization (FOIS 1–3) (Figure 1). 

### 3.1. Patient Characteristics

Table 1 shows the results of the comparison of patient characteristics across the three groups. Regarding the characteristics of the study participants, the median age was 85 years, and 66.9% of the patients had been living at home before hospitalization. Of the total 450 patients, 29 were in the Poor FOIS group, 189 in the Moderate FOIS group, and 232 in the Good FOIS group. Regarding FOIS scores from pre-admission to discharge, the scores decreased from 7 to 6 in the Good FOIS group and from 7 to 5 in the Moderate FOIS group, whereas in the Poor FOIS group, there was a marked decrease from 5 to 1. Compared to the Good FOIS group, the Poor FOIS group had a higher median age of 84 years and a higher proportion of men (79.3%). Furthermore, the Poor FOIS group had a higher proportion of severe and very severe cases on the A-DROP score, and the duration of intravenous antibiotic administration was significantly longer. The length of hospital stay was also significantly longer in the Poor FOIS group than in the other groups. Regarding the FIM, an indicator of ADL, scores were significantly lower in the Poor FOIS group at both the start of PR and at discharge. The proportions of patients living at home before hospitalization and after discharge were lower in the Poor and Moderate FOIS groups than in the Good FOIS group. 

Regarding ESM_CSA_/BSA, an indicator of muscle mass, the values were 9.95 cm^2^/m^2^ in the Poor FOIS group and 9.85 cm^2^/m^2^ in the Moderate FOIS group, whereas the Good FOIS group showed a significantly higher value of 11.64 cm^2^/m^2^. No significant differences were observed among the three groups with regard to ESM_CT_, an indicator of muscle quality.

### 3.2. Correlation Between FOIS at Discharge and ESM_CSA_/BSA and ESM_CT_

**Figure 2:**
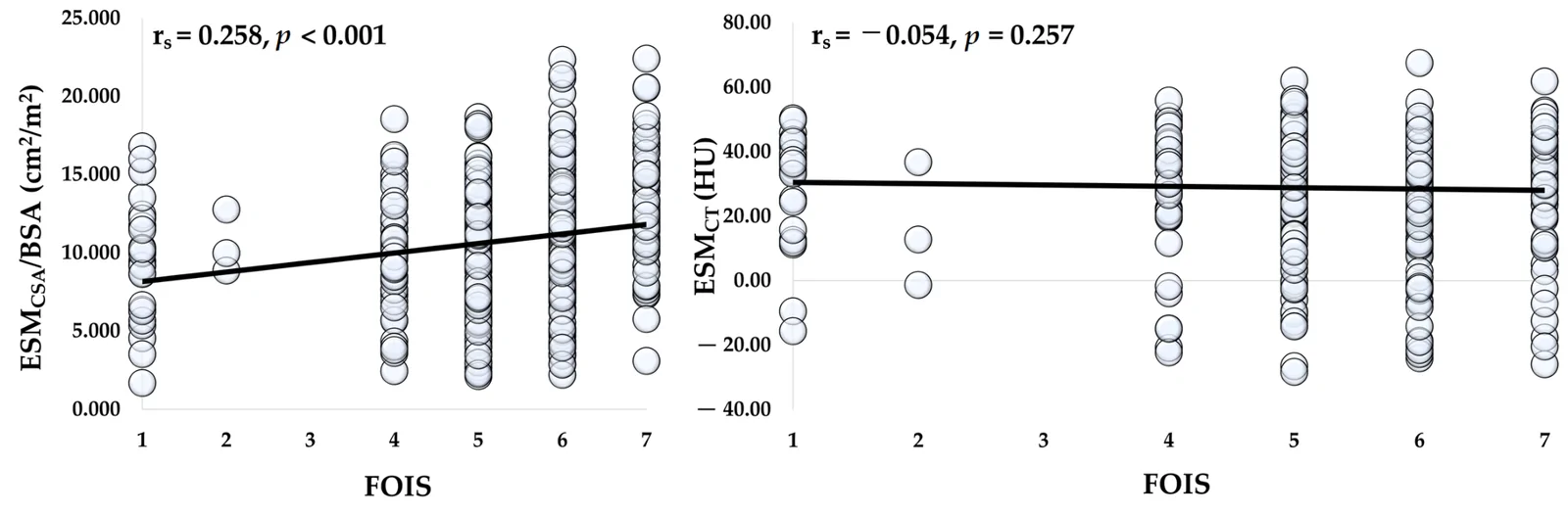
Correlation between FOIS at discharge and ESM_CSA_/BSA and ESM_CT_. The colored circles indicate individual patient observations, while the solid line indicates the fitted linear regression line. There was a significant positive correlation between FOIS at discharge and ESM_CSA_/BSA, a measure of muscle mass (r_s_ = 0.258, *p* < 0.001). There was no correlation between FOIS at discharge and ESM_CT_, a measure of muscle quality (r_s_ = −0.054, *p* = 0.257). BSA, body surface area; ESM_CSA_, cross-sectional area of the erector spinae muscles; ESM_CT_, mean radiographic density of the erector spinae muscles; FOIS, functional oral intake scale; HU, Hounsfield unit.

There was a significant positive correlation between FOIS at discharge and ESM_CSA_/BSA, a measure of muscle mass (r_s_ = 0.258, *p* < 0.001). In contrast, no significant correlation was found between FOIS at discharge and ESM_CT_, a measure of muscle quality (r_s_ = −0.054, *p* = 0.257) (Figure 2).

### 3.3. Results of an Ordinal Logistic Regression and Stratified Analysis with FOIS at Discharge as the Dependent Variable

**Figure 3:**
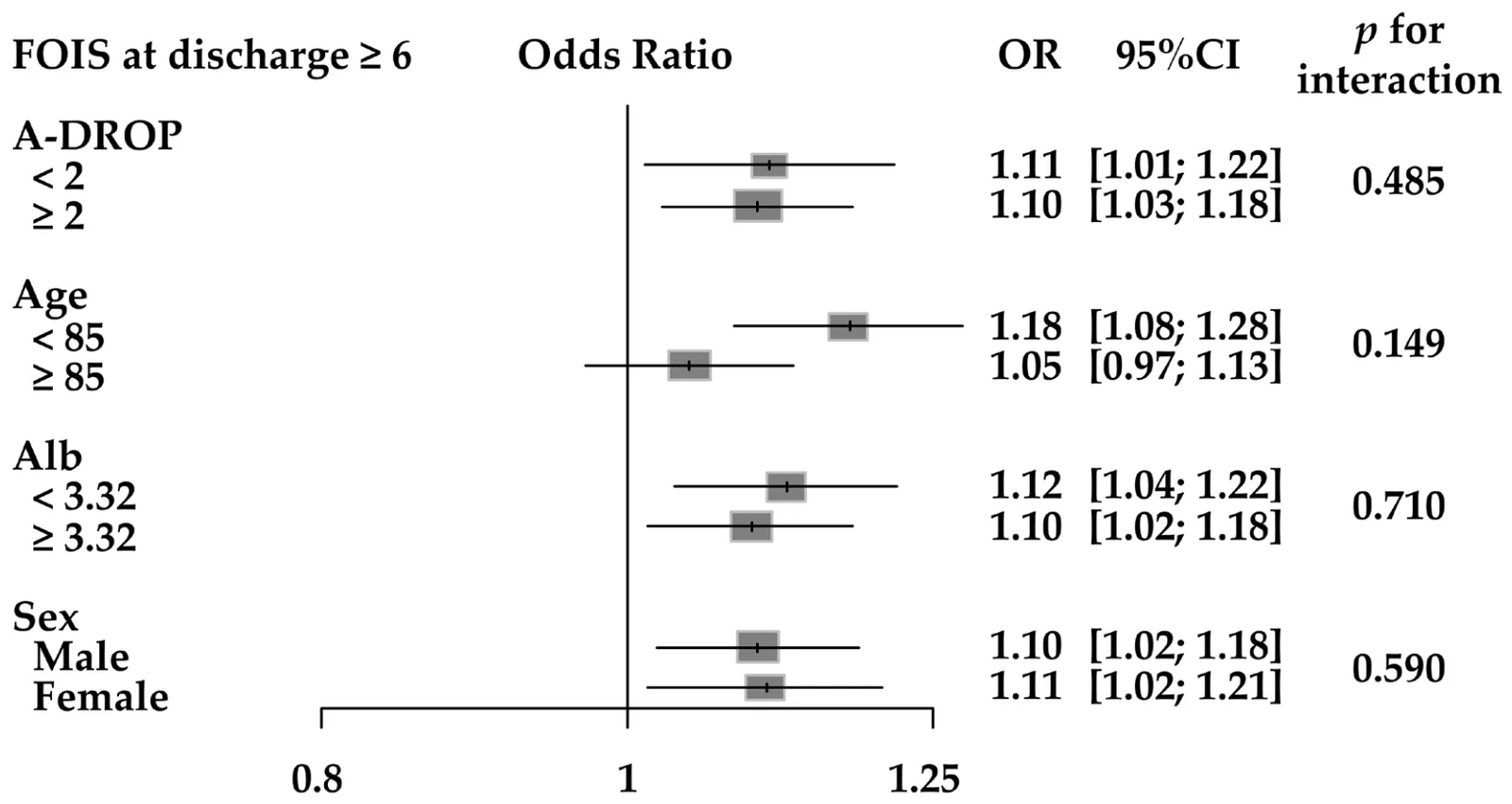
Forest plot of a stratified analysis on the association between ESM_CSA_/BSA and FOIS at discharge of ≥6. In the stratified analysis, an increase in ESM_CSA_/BSA was consistently associated with a FOIS at discharge of ≥6. Alb, albumin; BSA, body surface area; CI, confidence interval; ESM_CSA_, cross-sectional area of the erector spinae muscles; FOIS, functional oral intake scale; OR, odds ratio.

Table 2 shows the results of an ordered logistic regression analysis with FOIS at discharge as the dependent variable and ESM_CSA_/BSA and ESM_CT_ as explanatory variables. We confirmed that the VIF was 2 or less in both Model 1 and Model 2 after adjusting for confounding factors. In Model 1, which adjusted for confounding factors other than the FIM score at the start of PR, an indicator of muscle mass (ESM_CSA_/BSA) was shown to be independently associated with the FOIS score at discharge (OR: 1.09; 95% CI: 1.03–1.15; *p* = 0.004). However, in Model 2, which included FIM score at the start of PR, ESM_CSA_/BSA showed no association with FOIS at discharge (OR: 1.04; 95% CI: 0.98–1.11; *p* = 0.180). ESM_CT_ showed no significant association with FOIS at discharge in either Model 1 or Model 2. In the stratified analysis, an increase in ESM_CSA_/BSA was consistently associated with a FOIS score ≥ 6 at discharge across all subgroups, including age, sex, A-DROP, and Alb (Figure 3).

### 3.4. Results of Sensitivity Analysis in Patients with FOIS ≥ 6 Before Hospitalization

There were 378 patients with FOIS ≥ 6 before hospitalization. The study population was divided into two groups based on FOIS score ≥ 6 at discharge, and the results of the between-group comparisons are shown in Table 3. At discharge, 226 patients had a FOIS score of ≥6. The group with a FOIS score of ≥6 at discharge showed a significantly higher value of 11.66 cm^2^/m^2^ for ESM_CSA_/BSA, an indicator of muscle mass. No significant difference was observed between the two groups in terms of ESM_CT_, an indicator of muscle quality.

Table 4 shows the results of a multiple logistic regression analysis with FOIS score ≥ 6 at discharge as the dependent variable. We confirmed that the VIF was 2 or less in both Model 1 and Model 2, which were adjusted for confounding factors. In Model 1, which adjusted for confounding factors other than FIM score at the start of PR, an indicator of muscle mass (ESM_CSA_/BSA) was shown to be independently associated with FOIS at discharge (OR: 1.07; 95% CI: 1.01–1.14; *p* = 0.030). In Model 2, which included FIM score at the start of PR as a confounding factor, ESM_CSA_/BSA showed no association with FOIS at discharge (OR: 1.03; 95% CI: 0.96–1.10; *p* = 0.390). For ESM_CT_, an indicator of muscle quality, neither Model 1 nor Model 2 showed a significant association with FOIS at discharge. These results showed a similar trend to that observed before conducting sensitivity analyses.

## 4. Discussion

We examined the association between swallowing function at discharge and muscle mass (ESM_CSA_/BSA) and muscle quality (ESM_CT_), as measured using chest CT images, in older patients with pneumonia. The results showed that ESM_CSA_/BSA, an indicator of muscle mass, was associated with FOIS at discharge; however, this association disappeared after adjustment for FIM at the start of PR. In contrast, ESM_CT_, an indicator of muscle quality, showed no association with FOIS at discharge. These findings suggest that muscle mass, but not muscle quality, may be related to swallowing function in this population. This study demonstrates a potential association between swallowing function at discharge and CT-derived muscle mass in older patients with pneumonia. These results support the use of routinely obtained chest CT images to help identify patients at risk of dysphagia without the need for additional testing.

Recently, attention has been focused on the relationship between sarcopenia and swallowing function. Studies [5,8,24] have also shown that sarcopenia is independently associated with dysphagia. A position paper on sarcopenia and dysphagia published in 2019 also includes “systemic skeletal muscle loss” in its diagnostic algorithm, indicating that skeletal muscle loss is an important factor in dysphagia [25].

We focused on ESM because it can be consistently measured using chest CT scans—which are frequently performed for the diagnosis of pneumonia—and because there have been increasing reports of its association with physical function, respiratory function, and clinical outcomes in older adults. The ESMs examined in this study have several roles that are believed to contribute to physical function. First, the ESM is related to respiratory function. The ESM is one of the important respiratory accessory muscles, and previous studies have shown that ESM muscle mass measured using chest CT images is associated with respiratory function, such as forced expiratory volume in 1 s and vital capacity [11]. Furthermore, respiratory function is significantly associated with cough strength, which is considered important in preventing pneumonia [26], and individuals who developed aspiration pneumonia had significantly reduced cough strength [27]. There is also a close relationship between swallowing and respiratory function. A study of patients with chronic obstructive pulmonary disease reported a significant association between respiratory-swallowing desynchrony and respiratory function and the frequency of acute exacerbations [28]. These findings suggest that reduced ESM muscle mass may affect swallowing function through its impact on declining respiratory function. 

Second, the ESM is related to posture control. Swallowing and posture are closely related, and among the pathological background diseases leading to asymptomatic or overt aspiration, age-related skeletal changes, such as kyphosis and scoliosis, are also described [29]. The ESM is one of the critical postural muscles, and a decrease in the cross-sectional area and density of the thoracic trunk muscles is significantly associated with thoracic kyphosis [30].

Furthermore, trunk muscle mass has been reported to be significantly correlated with swallowing-related muscle mass [31]. Studies have reported a 31% prevalence of dysphagia among patients with sarcopenia [32]. Sarcopenia is significantly associated with the development of dysphagia during hospitalization [9,33]. Based on these findings, increased catabolism associated with the onset of pneumonia arises from the conditions of frailty and sarcopenia specific to the aging population. This increased catabolism leads to a further decrease in muscle mass, which in turn causes a decline in respiratory function and postural stability and affects swallowing function. Consequently, we suggest that this creates a vicious cycle—in which reduced energy intake leads to further muscle loss—thereby increasing the risk of swallowing dysfunction. Therefore, decreased ESM muscle mass measured using CT in this study may be associated with a decline in swallowing function during hospitalization.

Although decreased trunk muscle mass may not directly affect swallowing function, it can indirectly create unfavorable conditions for swallowing, which may contribute to the onset of aspiration pneumonia. Although FOIS remains the standard clinical measure of swallowing outcomes, CT-derived ESM assessment may provide complementary information, as chest CT imaging is frequently performed during the diagnostic workup of pneumonia. Therefore, ESM characteristics should not be considered a substitute for direct swallowing assessment but rather a potential complementary marker when CT images are already available.

However, in this study, ESM_CT_, an indicator of muscle quality, was not associated with swallowing dysfunction either alone or in combination, which differs from previous findings [10,14]. This finding may be due to the conditions and characteristics of the skeletal muscles measured. The ESM is generally considered to have a higher distribution of Type I fibers than Type II fibers [34], which is thought to favor muscle endurance rather than muscle strength. According to one study [10], the target muscle was the quadriceps femoris, which has a higher distribution of Type II fibers that favor muscle strength rather than muscle endurance, and this characteristic was considered to have a high correlation with the strength of the swallowing muscles. According to another study [14], muscle mass was measured at the level of the 12th thoracic vertebra, as in this study, but the difference between their findings and ours was likely due to their measurement of muscles other than the ESMs. In the future, it will be necessary to examine the effects of muscle quality on swallowing dysfunction in older patients with pneumonia, including muscles other than the ESM. 

Notably, the association between the ESM_CSA_/BSA and the FOIS score at discharge disappeared when the FIM score at the start of PR was adjusted. These findings suggest that muscle mass may partially reflect overall physical function rather than having a direct and independent effect on the recovery of swallowing function. Therefore, in patients with low muscle mass as measured by ESM, poor overall physical function may be one factor contributing to a poorer prognosis for swallowing function. However, physical function as assessed by the FIM may not merely be a confounding factor but may also function as a mediating variable in the causal pathway linking skeletal muscle mass to the recovery of swallowing function. Consequently, if physical function is positioned within the causal pathway between skeletal muscle mass and swallowing function recovery, adjustment for the FIM may have underestimated the apparent contribution of ESM_CSA_/BSA to swallowing outcomes. Because this study was a retrospective observational study, it was not possible to identify causal relationships among skeletal muscle mass, physical function, and swallowing function recovery. To elucidate these complex interrelationships, future prospective studies incorporating mediation analysis are needed.

Based on the above, assessment of ESM characteristics from chest CT images that are already obtained during routine pneumonia management may provide complementary information regarding swallowing outcomes.

This study has some limitations. First, this is a single-center study, and there are concerns regarding the applicability of the results to other facilities. Based on the results of the stratified analysis, which showed no age-related interaction, it is reasonable to conclude that ESM muscle mass is consistently associated with swallowing function at discharge; however, further multicenter studies are needed. Second, in this study, the relationship between muscle mass reduction and muscle quality deterioration in muscles involved in swallowing, which are directly related to swallowing function, remains unclear. Furthermore, because CT-based measures are difficult to perform on a regular basis due to radiation exposure risks, the results of this study do not support recommending the addition of new CT examinations. In the future, it will be necessary to proactively explore the use of minimally invasive assessment methods, such as ultrasound. Third, this study examined muscle mass and muscle quality at a single time point during hospitalization and did not assess skeletal muscle changes after hospitalization. Because skeletal muscle status is dynamic, particularly in older patients with pneumonia, a single measurement may not fully capture temporal changes occurring during recovery. Previous studies have shown that muscle mass, as measured by ESM on chest CT images, decreases during treatment in older patients with aspiration pneumonia [35]. Further studies are needed to determine whether longitudinal changes in ESM muscle mass and muscle quality are associated with swallowing outcomes and other clinically relevant endpoints. Fourth, this study focused on the ESM but did not examine other muscles. Therefore, it is necessary to examine other muscles as indicators to determine whether the results of this study apply to muscles beyond the ESM or whether they are specific to the ESM. Fifth, this study did not measure inflammatory cytokines such as Interleukin-6 and tumor necrosis factor-alpha. Future studies that incorporate these inflammatory biomarkers may help elucidate the biological mechanisms linking muscle loss to dysphagia and represent an area for further investigation. Finally, while this study used the FOIS to assess swallowing function, it did not perform physiological evaluations of swallowing using videofluoroscopic swallowing study (VFSS) or fiberoptic endoscopic evaluation of swallowing (FEES) examinations. Therefore, it is not possible to clearly distinguish whether the association between ESM and FOIS is due to swallowing function itself or is mediated by physical function or general health status. In future studies, it will be necessary to conduct a detailed evaluation of swallowing function using VFSS or FEES and then examine the association with ESM.

## 5. Conclusions

Lower CT-derived ESM mass was associated with poorer swallowing outcomes in older patients with pneumonia. However, this association was attenuated after adjustment for physical function, suggesting that ESM mass may primarily reflect overall physical function rather than act as an independent predictor of swallowing recovery. Further multicenter prospective studies are needed to clarify the underlying mechanisms and clinical utility of CT-derived muscle assessment.

## Figures and Tables

**Table 1:** Comparisons of patient characteristics in each group.

Variables		Overall	Poor FOIS Group (FOIS 1–3)	Moderate FOIS Group (FOIS 4–5)	Good FOIS Group (FOIS 6–7)	*p*-Value
		*n* = 450	*n* = 29	*n* = 189	*n* = 232	
Age	years	85.00 [78.00, 90.00]	84.00 [79.00, 88.00]	88.00 [82.00, 92.00]	82.00 [75.75, 87.25]	<0.001
Sex (male)	no (%)	255 (56.7)	23 (79.3)	102 (54.0)	130 (56.0)	0.036
BMI	kg/m^2^	20.59 [18.02, 23.32]	20.08 [17.33, 22.63]	20.41 [18.01, 23.42]	20.80 [18.26, 23.27]	0.482
CCI	score	2.00 [1.00, 3.00]	2.00 [1.00, 3.00]	2.00 [1.00, 3.00]	2.00 [1.00, 3.00]	0.198
A-DROP	score	2.00 [1.00, 2.00]	2.00 [1.00, 3.00]	2.00 [1.00, 2.00]	2.00 [1.00, 2.00]	<0.001
	mild, no (%)	20 (4.4)	0 (0.0)	4 (2.1)	16 (6.9)	0.001
	moderate, no (%)	344 (76.4)	19 (65.5)	138 (73.0)	187 (80.6)	
	severe, no (%)	71 (15.8)	7 (24.1)	38 (20.1)	26 (11.2)	
	very severe, no (%)	15 (3.3)	3 (10.3)	9 (4.8)	3 (1.3)	
Mechanical ventilation	no (%)	5 (1.1)	0 (0.0)	2 (1.1)	3 (1.3)	0.818
Intravenous antibiotic administration	days	9.00 [7.00, 13.00]	14.00 [11.00, 23.00]	9.00 [7.00, 13.00]	9.00 [6.00, 11.25]	<0.001
Oral or EN within 48 h	no (%)	363 (80.7)	11 (37.9)	135 (71.4)	217 (93.5)	<0.001
PR within 48 h	no (%)	261 (58.0)	21 (72.4)	103 (54.5)	137 (59.1)	0.171
WBC	×10^2^/μL	99.00 [71.00, 130.00]	105.00 [69.00, 145.00]	98.00 [71.00, 131.00]	98.50 [70.75, 129.00]	0.8
CRP	mg/dL	7.48 [2.46, 13.70]	7.12 [2.52, 12.90]	6.31 [2.39, 12.72]	8.10 [2.59, 14.50]	0.473
Alb	g/dL	3.32 [2.97, 3.63]	3.03 [2.80, 3.36]	3.26 [2.84, 3.58]	3.38 [3.03, 3.67]	0.001
Hb	g/dL	11.60 [10.33, 12.90]	11.70 [10.50, 13.20]	11.50 [9.90, 12.80]	11.70 [10.70, 12.90]	0.083
FIM score at the start of PR	points	67.00 [39.00, 93.00]	30.00 [21.00, 35.00]	45.00 [30.00, 68.00]	89.00 [68.00, 103.00]	<0.001
FIM score at discharge	points	85.00 [47.00, 109.00]	23.00 [21.00, 34.00]	60.00 [35.00, 83.00]	105.00 [88.75, 118.00]	<0.001
FOIS score before hospitalization	score	7.00 [6.00, 7.00]	5.00 [5.00, 6.00]	7.00 [5.00, 7.00]	7.00 [7.00, 7.00]	<0.001
FOIS score at discharge	score	6.00 [5.00, 6.00]	1.00 [1.00, 1.00]	5.00 [4.00, 5.00]	6.00 [6.00, 7.00]	<0.001
ESM_CSA_/BSA	cm^2^/m^2^	10.72 [7.89, 13.38]	9.95 [6.66, 11.71]	9.85 [7.30, 12.74]	11.64 [9.22, 14.20]	<0.001
ESM_CT_	HU	33.34 [20.23, 41.52]	36.28 [15.65, 41.51]	33.95 [20.85, 41.86]	32.61 [20.16, 40.95]	0.666
Residence location before hospitalization	Home, no (%)	301 (66.9)	9 (31.0)	100 (52.9)	192 (82.8)	<0.001
Residence location after hospital discharge	Home, no (%)	264 (58.7)	4 (13.8)	79 (41.8)	181 (78.0)	<0.001
Length of hospital stay	days	21.00 [12.00, 37.75]	64.00 [42.00, 78.00]	24.00 [14.00, 42.00]	15.00 [10.00, 26.00]	<0.001

Data are presented as median (25th–75th percentiles) or number (percentage). Statistical analyses were performed using the Kruskal–Wallis test and the chi-square (χ^2^) test. Alb: albumin; BMI: body mass index; BSA: body surface area; CCI: Charlson Comorbidity Index; CRP: C-reactive protein; EN: enteral nutrition; ESM_CSA_: cross-sectional area of the erector spinae muscles; ESM_CT_: mean radiographic density of the erector spinae muscles; FIM: functional independence measure; FOIS: functional oral intake scale; Hb: hemoglobin; HU: Hounsfield unit; PR: pulmonary rehabilitation; WBC: white blood cell.

**Table 2:** Results of an ordinal logistic regression analysis with FOIS at discharge as the dependent variable.

Variables	Model 1			Model 2		
	OR (95% CI)	*p*-Value	VIF	OR (95% CI)	*p*-Value	VIF
Age	0.96 (0.93–0.99)	0.014	1.242	0.98 (0.95–1.01)	0.229	1.283
Sex (female)	1.71 (1.10–2.68)	0.018	1.195	2.00 (1.25–3.24)	0.004	1.202
BMI	1.00 (0.95–1.05)	0.997	1.035	1.02 (0.97–1.08)	0.466	1.045
CCI	0.95 (0.81–1.10)	0.465	1.056	0.97 (0.82–1.14)	0.675	1.080
A-DROP	0.78 (0.61–0.99)	0.040	1.085	1.01 (0.78–1.31)	0.960	1.145
CRP	1.03 (1.00–1.06)	0.046	1.157	1.02 (0.99–1.06)	0.118	1.175
Alb	1.29 (0.86–1.95)	0.216	1.177	1.14 (0.74–1.75)	0.556	1.192
Intravenous antibiotic administration	0.94 (0.92–0.97)	<0.001	1.066	0.96 (0.93–0.99)	0.005	1.078
FOIS score before hospitalization	3.36 (2.55–4.50)	<0.001	1.050	2.38 (1.77–3.23)	<0.001	1.093
FIM score at the start of PR	-	-	-	1.04 (1.03–1.05)	<0.001	1.261
ESM_CSA_/BSA	1.09 (1.03–1.15)	0.004	1.174	1.04 (0.98–1.11)	0.180	1.206
ESM_CT_	1.00 (0.98–1.01)	0.460	1.012	1.00 (0.99–1.01)	0.706	1.030

Model 1 was adjusted for age, sex, BMI, CCI, A-DROP, CRP, Alb, intravenous antibiotic administration, and FOIS score before hospitalization. Model 2 was adjusted for age, sex, BMI, CCI, A-DROP, CRP, Alb, intravenous antibiotic administration, and the FOIS score before hospitalization, with the addition of the FIM score at the start of PR. Alb: albumin; BMI: body mass index; BSA: body surface area; CCI: Charlson Comorbidity Index; CI: confidence interval; CRP: C-reactive protein; ESM_CSA_: cross-sectional area of the erector spinae muscles; ESM_CT_: mean radiographic density of the erector spinae muscles; FIM: functional independence measure; FOIS: functional oral intake scale; OR: odds ratio; PR: pulmonary rehabilitation; VIF: variance inflation factor.

**Table 3:** Comparisons of patient characteristics in patients who had a FOIS score ≥ 6 before hospitalization (*n* = 378).

Variables		FOIS at Discharge		*p*-Value
		<6 (*n* = 152)	≥6 (*n* = 226)	
Age	years	87.50 [81.00, 91.00]	82.00 [75.00, 87.75]	<0.001
Sex (male)	no (%)	86 (56.6)	127 (56.2)	1.000
BMI	kg/m^2^	20.41 [17.77, 23.44]	20.62 [18.08, 23.15]	0.745
CCI	score	2.00 [1.00, 3.00]	2.00 [1.00, 2.75]	0.459
A-DROP	score	2.00 [1.00, 3.00]	2.00 [1.00, 2.00]	<0.001
	mild, no (%)	3 (2.0)	16 (7.1)	0.001
	moderate, no (%)	109 (71.7)	182 (80.5)	
	severe, no (%)	33 (21.7)	25 (11.1)	
	very severe, no (%)	7 (4.6)	3 (1.3)	
Mechanical ventilation	no (%)	2 (1.3)	3 (1.3)	1.000
Intravenous antibiotic administration	days	9.00 [7.00, 14.00]	9.00 [7.00, 12.00]	0.051
Oral or EN within 48 h	no (%)	114 (75.0)	212 (93.8)	<0.001
PR within 48 h	no (%)	88 (57.9)	133 (58.8)	0.938
WBC	×10^2^/μL	98.00 [69.00, 129.00]	98.50 [70.25, 129.00]	0.956
CRP	mg/dL	6.25 [2.44, 11.86]	8.10 [2.57, 14.53]	0.183
Alb	g/dL	3.30 [2.86, 3.61]	3.40 [3.03, 3.68]	0.058
Hb	g/dL	11.70 [10.20, 13.20]	11.70 [10.70, 12.90]	0.401
FIM score at the start of PR	points	48.00 [33.75, 73.75]	89.50 [68.00, 103.00]	<0.001
FIM score at discharge	points	62.00 [36.75, 85.00]	105.00 [89.00, 118.75]	<0.001
FOIS score before hospitalization	score	7.00 [6.00, 7.00]	7.00 [7.00, 7.00]	<0.001
FOIS score at discharge	score	5.00 [4.75, 5.00]	6.00 [6.00, 7.00]	<0.001
ESM_CSA_/BSA	cm^2^/m^2^	9.93 [7.01, 13.04]	11.66 [9.23, 14.26]	<0.001
ESM_CT_	HU	35.17 [21.18, 42.36]	32.61 [19.96, 40.98]	0.201
Residence location before hospitalization	Home, no (%)	93 (61.2)	188 (83.2)	<0.001
Residence location after hospital discharge	Home, no (%)	73 (48.0)	177 (78.3)	<0.001
Length of hospital stay	days	24.50 [13.00, 46.00]	15.00 [10.00, 25.75]	<0.001

Data are presented as median (25th–75th percentiles) or number (percentage). Statistical analyses were performed using the Mann–Whitney U test and the chi-square (χ^2^) test. Alb: albumin; BMI: body mass index; BSA: body surface area; CCI: Charlson Comorbidity Index; CRP: C-reactive protein; EN: enteral nutrition; ESM_CSA_: cross-sectional area of the erector spinae muscles; ESM_CT_: mean radiographic density of the erector spinae muscles; FIM: functional independence measure; FOIS: functional oral intake scale; Hb: hemoglobin; HU: Hounsfield unit; PR: pulmonary rehabilitation; WBC: white blood cell.

**Table 4:** Sensitivity analysis of a multiple logistic regression analysis using the FOIS score at discharge as the dependent variable in patients who had a FOIS score of ≥6 before hospitalization (*n* = 378).

Variables	Model 1			Model 2		
	OR (95% CI)	*p*-Value	VIF	OR (95% CI)	*p*-Value	VIF
Age	0.95 (0.91–0.98)	0.001	1.209	0.96 (0.92–1.00)	0.032	1.238
Sex (female)	1.41 (0.84–2.35)	0.189	1.243	1.59 (0.91–2.77)	0.103	1.257
BMI	1.00 (0.95–1.06)	0.920	1.046	1.03 (0.97–1.09)	0.387	1.067
CCI	0.99 (0.83–1.17)	0.891	1.068	1.01 (0.84–1.21)	0.939	1.088
A-DROP	0.75 (0.56–1.00)	0.047	1.127	0.98 (0.72–1.35)	0.917	1.198
CRP	1.03 (1.00–1.07)	0.038	1.153	1.03 (1.00–1.07)	0.070	1.169
Alb	1.43 (0.89–2.29)	0.139	1.164	1.35 (0.81–2.25)	0.254	1.180
Intravenous antibiotic administration	0.98 (0.95–1.01)	0.110	1.061	1.00 (0.97–1.03)	0.862	1.093
FOIS score before hospitalization	4.03 (2.05–7.93)	<0.001	1.021	2.47 (1.18–5.15)	0.016	1.044
FIM score at the start of PR	-	-	-	1.04 (1.03–1.05)	<0.001	1.271
ESM_CSA_/BSA	1.07 (1.01–1.14)	0.030	1.192	1.03 (0.96–1.10)	0.390	1.230
ESM_CT_	1.00 (0.98–1.01)	0.516	1.010	1.00 (0.99–1.02)	0.729	1.039

Model 1 was adjusted for age, sex, BMI, CCI, A-DROP, CRP, Alb, intravenous antibiotic administration, and FOIS score before hospitalization. Model 2 was adjusted for age, sex, BMI, CCI, A-DROP, CRP, Alb, intravenous antibiotic administration, and the FOIS score before hospitalization, with the addition of the FIM score at the start of PR. The AUC for Model 1 was 0.740 (95% CI: 0.690–0.790). The AUC for Model 2 was 0.816 (95% CI: 0.772–0.860). Alb: albumin; AUC: area under the receiver operating characteristic curve; BMI: body mass index; BSA: body surface area; CCI: Charlson Comorbidity Index; CI: confidence interval; CRP: C-reactive protein; ESM_CSA_: cross-sectional area of the erector spinae muscles; ESM_CT_: mean radiographic density of the erector spinae muscles; FIM: functional independence measure; FOIS: functional oral intake scale; OR: odds ratio; PR: pulmonary rehabilitation; VIF: variance inflation factor.

## Data Availability

The data collected/analyzed in the current study have not been released to the public for privacy reasons. If there is a reasonable request, we will consider submitting the data.

## Abbreviations

Alb	Albumin
BMI	Body mass index
BSA	Body surface area
CCI	Charlson Comorbidity Index
CI	Confidence interval
CRP	C-reactive protein
CT	Computed tomography
EN	Enteral nutrition
ESM	Erector spinae muscles
ESM_CSA_	Cross-sectional area of the erector spinae muscles
ESM_CT_	Mean radiographic density of the erector spinae muscles
FIM	Functional independence measure
FOIS	Functional oral intake scale
Hb	Hemoglobin
HU	Hounsfield unit
OR	Odds ratio
PR	Pulmonary rehabilitation
ROC	Receiver operating characteristic
WBC	White blood cell
